# A population study of the minicircles in *Trypanosoma cruzi*: predicting guide RNAs in the absence of empirical RNA editing

**DOI:** 10.1186/1471-2164-8-133

**Published:** 2007-05-24

**Authors:** Sean Thomas, LL Isadora Trejo Martinez, Scott J Westenberger, Nancy R Sturm

**Affiliations:** 1 Molecular Biology Institute, UCLA, Los Angeles, CA, USA; 2 Department of Microbiology, Immunology and Molecular Genetics, UCLA, Los Angeles, CA, USA

## Abstract

**Background:**

The structurally complex network of minicircles and maxicircles comprising the mitochondrial DNA of kinetoplastids mirrors the complexity of the RNA editing process that is required for faithful expression of encrypted maxicircle genes. Although a few of the guide RNAs that direct this editing process have been discovered on maxicircles, guide RNAs are mostly found on the minicircles. The nuclear and maxicircle genomes have been sequenced and assembled for *Trypanosoma cruzi*, the causative agent of Chagas disease, however the complement of 1.4-kb minicircles, carrying four guide RNA genes per molecule in this parasite, has been less thoroughly characterised.

**Results:**

Fifty-four CL Brener and 53 Esmeraldo strain minicircle sequence reads were extracted from *T. cruzi *whole genome shotgun sequencing data. With these sequences and all published *T. cruzi *minicircle sequences, 108 unique guide RNAs from all known *T. cruzi *minicircle sequences and two guide RNAs from the CL Brener maxicircle were predicted using a local alignment algorithm and mapped onto predicted or experimentally determined sequences of edited maxicircle open reading frames. For half of the sequences no statistically significant guide RNA could be assigned. Likely positions of these unidentified gRNAs in *T. cruzi *minicircle sequences are estimated using a simple Hidden Markov Model. With the local alignment predictions as a standard, the HMM had an ~85% chance of correctly identifying at least 20 nucleotides of guide RNA from a given minicircle sequence. Inter-minicircle recombination was documented. Variable regions contain species-specific areas of distinct nucleotide preference. Two maxicircle guide RNA genes were found.

**Conclusion:**

The identification of new minicircle sequences and the further characterization of all published minicircles are presented, including the first observation of recombination between minicircles. Extrapolation suggests a level of 4% recombinants in the population, supporting a relatively high recombination rate that may serve to minimize the persistence of gRNA pseudogenes. Characteristic nucleotide preferences observed within variable regions provide potential clues regarding the transcription and maturation of *T. cruzi *guide RNAs. Based on these preferences, a method of predicting *T. cruzi *guide RNAs using only primary minicircle sequence data was created.

## Background

Kinetoplastids are single-celled protists, some of which are free-living, while *Trypanosoma cruzi *and others cause significant plant, animal, and human diseases. Decades after surviving the potentially deadly acute phase of Chagas disease, a human infected with *T. cruzi *may develop fatal damage to cardiac and smooth muscle tissue in approximately 30% of cases. Chagas disease may be caused by tissue destruction caused by the parasite, errant autoimmune responses or some combination of the two [1,2]. Chagas disease affects millions in South and Central America, and there are no predictive tests for disease outcome.

Among eukaryotes that possess mitochondrial genomes, there is a remarkable diversity of genome structure ranging from single circular chromosomes to extremely complex arrangements with multiple chromosomes found in organisms like *Amoebidium parasiticum *[3]. The mitochondrial DNA (kDNA) of the kinetoplastids is a unique structure comprised of dozens of ~25-Mb maxicircles and thousands of 1.4-kb minicircles linked together in a dense network called the kinetoplast. Each maxicircle copy is thought to be nearly identical, although this assumption may be incorrect [4], and the number of minicircle sequence classes ranges from one to over a hundred depending on the kinetoplastid. This structural behemoth belies a functional complexity whereby messages transcribed from maxicircles must be decrypted by means of a uridine insertion/deletion RNA editing process [5]. Following the hybridization of the 5'-anchor region of a guide RNA (gRNA) to the 3' end of its target message, sequential base pairing directs U insertion and deletion in a processive enzyme cascade [6]. Non-canonical G-U base pairs are permissible in these RNA-RNA interactions, conferring transition tolerance to the gRNA sequence, with a staggering number of potential guides directing identical editing events. Editing events cannot be predicted based solely on a gRNA sequence, nor *visa versa*. The primary repository of gRNAs are the minicircles [7], with a handful of gRNAs found on maxicircles [8].

In *T. cruzi *the kinetoplast DNA has two tantalizing links to disease. Minicircles can integrate into the host genome [9,10] potentially persisting long after an active infection has been cleared. This example of horizontal DNA transfer has implications for the autoimmune characteristics seen in the clinics. Parasite integration events have been localized to host LINE-1 retrotransposable elements, thus conferring mobility upon the parasite sequences as hundreds of thousands of these elements exist per genome [11]. Maxicircles may also play a role in pathology, as a lesion discovered in a parasite mitochondrial gene was correlated with disease presentation [12]. The extensively edited NADH dehydrogenase 7 (*ND7*) gene harbours a deletion that would compromise the electron transport chain, a defect found exclusively in *T. cruzi *strains isolated from asymptomatic patients. This and other related loci may provide the first functional linkage between parasite genotype and disease manifestation. The minicircle and maxicircle phenomena are not mutually exclusive. The potential association of these events with Chagas pathology makes understanding the structure and function of mitochondrial DNA particularly relevant in *T. cruzi*.

*T. cruzi *is divided into several strains, each with distinct geographic distributions, host preferences and disease severity [13-16]. These 'discrete typing units' (DTUs) delineate six subtypes (I, IIa, IIb, IIc, IId and IIe). DTUs IIb and I represent the ancestral *T. cruzi *lineages. Genetic recombination in *T. cruzi *occurs through a loosely-defined, whole-cell fusion mechanism [17]. The extant population structure can be derived from two discrete fusion events [18]. A fusion of DTUs I and IIb generated a hybrid ancestor to DTUs IIa and IIc that has shared elements of recombination and homozygosity and have since diverged from one another. A more recent DTU IIc/IIb fusion gave rise to the largely heterozygous DTUs IId and IIe subtypes. The CL Brener strain chosen for genome sequencing [19] belongs to DTU IIe, and Esmeraldo strain was sequenced to a lesser degree as a representative of DTU IIb. The maxicircle genomes fall into three distinct clades that are partitioned in among DTU I, DTU IIb, and DTUs IIa/IIc/IId/IIe [20,21]. The CL Brener and Esmeraldo maxicircles have been assembled in their entirety [22].

This report details the isolation of minicircle sequences from the whole genome shotgun (WGS) reads from the CL Brener and Esmeraldo strains of *T. cruzi *generated as a by-blow of the Genome Sequence Project [18]. In combination with the predicted or actual sequences of edited messages, a thorough characterization of all available *T. cruzi *minicircle data is presented. The features of the assigned gRNAs were then used to generate a selection scheme for gRNA genes in the absence of known editing events. Minicircles were assembled from the genome project sequence reads, revealing two instances of apparent minicircle recombination.

## Results

### Intragenomic conservation of conserved sequence blocks

A typical *T. cruzi *minicircle is approximately 1.4-kb and contains four conserved sequence regions, each followed by a variable region containing a gRNA. Each conserved region is composed of three individual conserved sequence blocks (CSB-1, CSB-2, and CSB-3) each of which are broadly conserved [23]. Numerous minicircle sequences with CSBs were found in the CL Brener and Esmeraldo strain WGS reads. Multicopy sequences can have extensive variability in kinetoplastids [24], so to be certain of isolating the greatest number of minicircle sequences, the intragenomic diversity of individual CSB sequences was first assessed by examining the conserved regions of all extracted *T. cruzi *minicircle sequences using a two-tag method to capture native variability of the intermediate sequence (see Methods).

**Figure 1:**
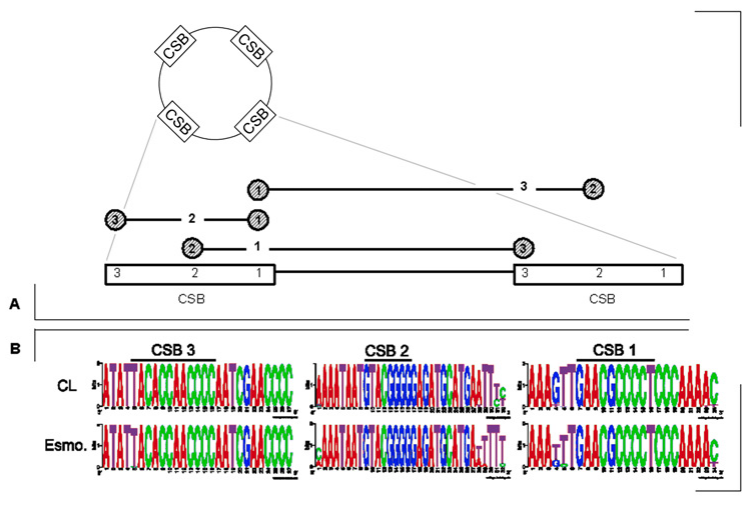
**Intragenomic conserved region conservation in minicircles**. A) Variation of CSB-1, CSB-2, and CSB-3 was assessed by using two static tags to pull out the natural variability of the third. B) Weblogo diagrams  show the high degree of intragenomic CSB conservation. At each position the height of the letter represents the proportion of sequences with the base represented by that letter

Remarkable conservation was observed among the dozens of conserved region sequences identified (Fig 1). Less than 1% of sequences contained variations at each position within the CSBs, and the conservation for each strain extended well beyond the basic consensus. The base CSB-1 sequence was expanded from 10 nt to 18 nt common to CL Brener and Esmeraldo minicircles, with a strong 24-nt stretch in CL Brener. The G-rich, 8-bp CSB-2 element is expanded into a 26-nt purine-rich region in both *T. cruzi *strains, with a 29 nt length in CL Brener. The 12-nt CSB-3 was extended to 27 nt in both strains. These extended CSBs likely reflect species-specificity and could prove useful for taxonomic distinctions.

The conserved regions of the minicircles represent tags that can be used to pull minicircle sequences from the WGS reads while being confident that few, if any, sequences are missed due to intragenomic variation.

### Minicircle sequences extracted from *T. cruzi *WGS reads

The areas of complete CSB conservation were used as tags to extract as many minicircle reads as possible. A sorting script extracted 54 CL Brener and 53 Esmeraldo sequence reads. If a sequence was found to contain minicircle sequence, then the read from that same clone in the opposite direction was also extracted from the database, and if the 'mate pair' sequences overlapped they were joined. In this manner 32 contiguous sequences were assembled. Dozens of the reads contained several complete variable regions, with each variable region potentially carrying a gRNA. The structural linkages between variable regions were recorded to determine if recombination occurred between minicircle sequence classes and if any correlation existed between editing target and minicircle neighbours.

**Figure 2:**
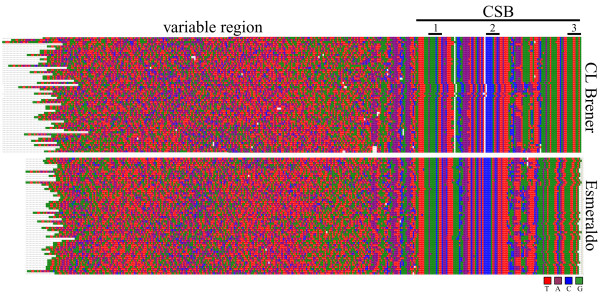
**Alignments of CL Brener and Esmeraldo minicircle variable regions**. Spectrogram representation of the CLUSTALX alignment of minicircle sequences identified from the CL Brener (top) and Esmeraldo (bottom) WGS reads. Each base within each read is represented as a coloured square ('T'-red; 'A'-purple; 'C'-blue; 'G'-green).

Each variable region was expected to encode a single gRNA, thus for the task of gRNA prediction the sequences were broken into individual units of the upstream CSB-3 plus one variable region plus its downstream CSB. A spectrogram alignment of unique units revealed striking regions of nucleotide preference in several places within the variable regions (Fig 2). An enrichment of G residues was evident adjacent to the CSBs, while the central portion of the variable region indicated a bias toward As and Cs. This pattern held true for both CL Brener and Esmeraldo strains.

The minicircle variable regions displayed specific nucleotide preferences over their length, potentially reflecting the sequence bias of the gRNA genes. We next sought to correlate the putative gRNA genes with RNA editing events, however as many of these sequences are undetermined, some creative sequence manipulation was required in order to proceed.

### Editing in *T. cruzi*

One of the major goals of this analysis is to predict gRNAs. The current local alignment method of predicting gRNAs [8] requires knowledge of the sequence for the fully edited target genes that for the most part are not yet known for *T. cruzi*, ATPase 6 [25] and COII [26] being the only exceptions. We have initiated the experimental determination of *T. cruzi *editing events, but in the meantime used predictions to facilitate our minicircle study.

Predicted edited sequences (Additional File 1) were generated by manually inserting or deleting Us in the unedited message sequence following the known sequences of the corresponding *T. brucei *edited mRNAs, while preserving conservation of the resulting amino acid sequence. Predicted sequences were generated for: CyB, CR3, CR4, MURF2, ND3, ND4, ND7, ND8, ND9, and RPS12. Concurrently, the edited sequence of COIII was obtained by RT-PCR for CL Brener: When compared with our *in silico *prediction, the true COIII sequence differed from the predicted version such that two of the estimated 30 gRNAs would have been missed (data not shown). For this reason, sizeable portions of the other predicted sequences were expected to yield many useful gRNA predictions. Additional confidence was gained for segments of predicted sequence where high-scoring matches with putative gRNAs were found.

A combination of actual and predicted mRNA sequences was used for exploring the gRNA assignments to specific genes. This method is not perfect due to the potential for error in our predictions, but these areas will be clarified as the edited sequences are obtained at the bench.

### *T. cruzi *gRNAs identified in minicircle and maxicircle sequences

**Figure 3:**
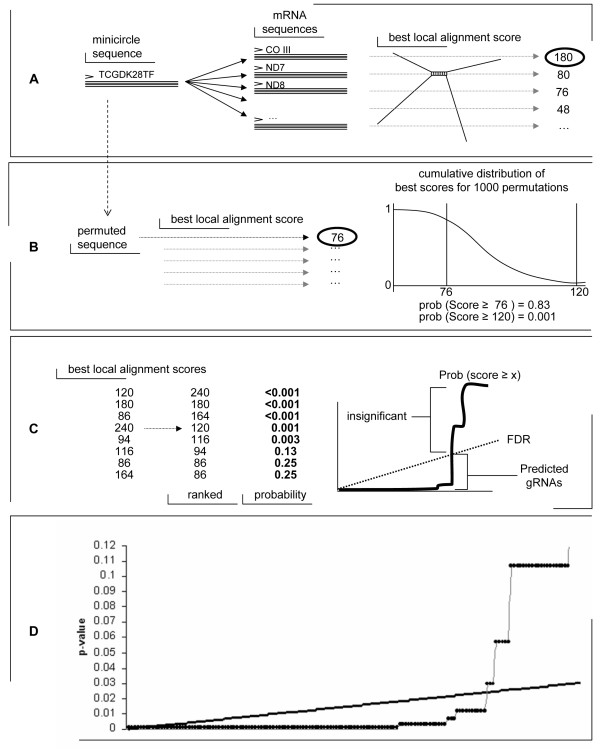
**gRNA identification method and validation**. A) A local alignment was performed for each minicircle sequence against a library of predicted edited mRNA sequences yielding a single best score. B) 1000 permutations and local alignment batteries were performed as a method of calculating the approximate probability of a given best score. C) The best hybridization scores for each minicircle sequence were ranked by cumulative probability from 0 to 1 (circles). All points to the left of the intersection with the false discovery rate threshold (heavy solid line) were deemed to represent scores from predicted gRNAs. D) Using false discovery rate to control for multiple testing, alignments with scores deemed significant were said to be predicted gRNAs.

Potential gRNAs were identified among minicircle sequences from the genome project and in GenBank using a three-part process (Fig. 3abc): the single greatest Smith-Waterman local alignment score for a variable region was most likely to represent the overlap of the gRNA and target mRNA region. A permutation test was performed to determine the probability of a given 'best overlap', and then a false discovery rate (FDR – see Methods) control was used to determine whether a hybridization with a given probability was significant (Fig. 3c). This statistic provided the criterion used to identify 108 potential gRNAs from 248 minicircle variable regions obtained from GenBank [25,27], CL Brener and Esmeraldo WGS reads (Additional File 2). The predicted minicircle gRNAs are positioned consistently within the variable region, providing more evidence that these highly scoring alignments represent regions of gRNA hybridization to target mRNAs.

**Figure 4:**
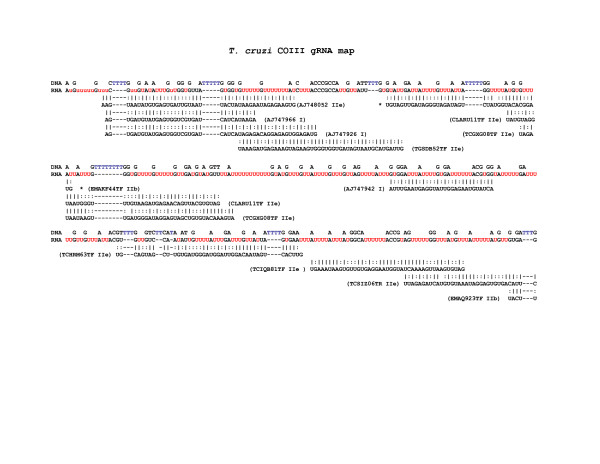
**COIII gRNA map for *T. cruzi***. Beneath the unedited COIII sequence, predicted gRNAs are mapped onto the fully edited CL Brener COIII mRNA sequence. Beside each putative gRNA is the WGS clone name or GenBank sequence identifier followed by the DTU strain designation for that sequence. Watson-Crick base pairs, '|', and non-canonical G-U base pairs, ':' are indicated.

**Figure 5:**
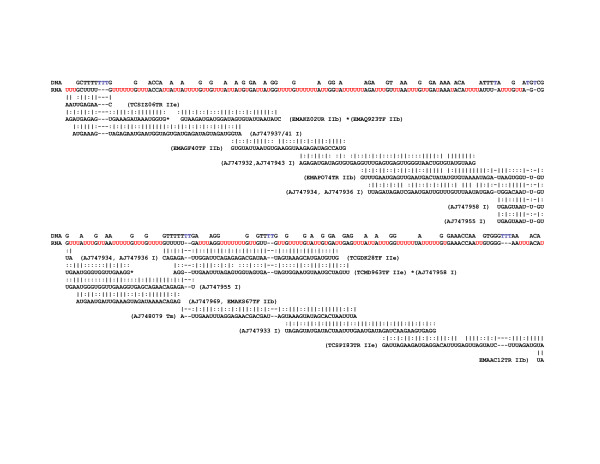
**COIII gRNA map for *T. cruzi***. Beneath the unedited COIII sequence, predicted gRNAs are mapped onto the fully edited CL Brener COIII mRNA sequence. Beside each putative gRNA is the WGS clone name or GenBank sequence identifier followed by the DTU strain designation for that sequence. Watson-Crick base pairs, '|', and non-canonical G-U base pairs, ':' are indicated.

**Figure 6:**
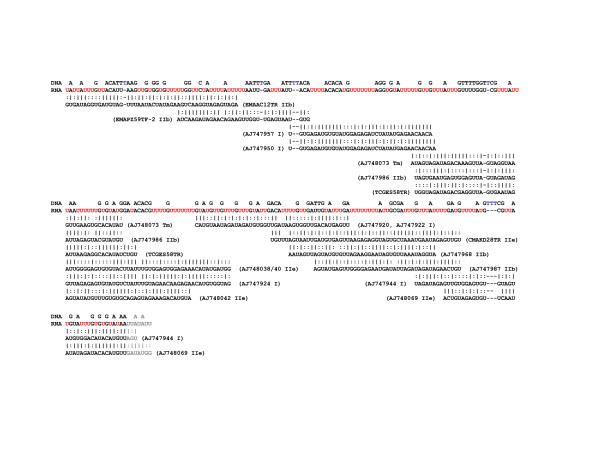
**COIII gRNA map for *T. cruzi***. Beneath the unedited COIII sequence, predicted gRNAs are mapped onto the fully edited CL Brener COIII mRNA sequence. Beside each putative gRNA is the WGS clone name or GenBank sequence identifier followed by the DTU strain designation for that sequence. Watson-Crick base pairs, '|', and non-canonical G-U base pairs, ':' are indicated.

The predicted maps of RNA editing for *T. cruzi *(Additional File 3) showed that while the final edited mRNA was conserved due to the required amino acid composition of the resulting proteins the gRNAs that perform the editing were variable from one strain to the next; this variability was not restricted to transition mutations. The inexact line-up of gRNAs from different strains on certain regions of the COIII map (Fig 4, 5, 6), for example, demonstrated that the start and stop positions of hybridization can drift from strain to strain, and that gRNA heterogeneity may occur within isolates.

In addition to gRNAs identified from minicircle sequences, two gRNAs were predicted by local alignments with high confidence to reside within CL Brener maxicircles. Both maxicircle gRNAs are on the opposite strand of nearby protein-coding genes, with an ND7 gRNA placed 95-bp downstream of ND5 and approximately 500-bp upstream of the repetitive region, and a MURF 2 gRNA located 66-bp downstream of the CR4 gene overlapping the start codon of ND4. The previously-described COII gRNA lies immediately downstream of the COII gene on the same coding strand [25,27].

### Areas of nucleotide preference within minicircle variable regions

The spectrograms of full minicircle units (CSB to CSB) revealed areas of conserved nucleotide preference within the variable region (Fig 2). To explore this further, the sequences with identified gRNAs were aligned as follows: regions upstream of the gRNAs were aligned to their 3' ends, gRNAs were all aligned at +1, 5' to 3' of predicted hybridization, and regions downstream of the gRNAs were aligned by their 5' ends.

**Figure 7:**
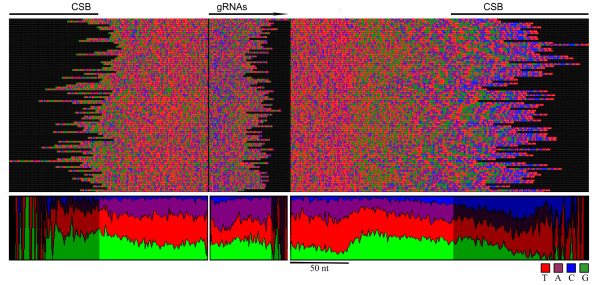
***T. cruzi *minicircle gRNA alignments reveal sequence bias**. Sequences containing predicted gRNAs were aligned by the boundaries of the hybridization prediction. A) Spectrogram ('T'-red; 'A'-purple; 'C'-blue; 'G'-green) of aligned minicircle sequences. B) Ratios of nucleotide composition ('T'-red; 'A'-purple; 'C'-blue; 'G'-green) of aligned sequences by position.

The resulting spectrogram revealed nucleotide preferences with respect to these orientations (Fig 7). As might be expected to promote annealing to the target message, the 5'-anchor region of the gRNAs had a higher than background preference for Cs (blue), and the entire gRNA had a slightly higher T to A skew (red to purple) with a spike centred approximately 12 nt into the predicted gRNA hybridization. While the areas immediately around the gRNAs were more T-rich (red) than the gRNAs themselves, the most clear transition occurred approximately 50-nt downstream of the gRNA where the preference for As and Ts dropped sharply and the region became more G-rich (green).

Given that the precise switch was dictated more by distance from the end of predicted hybridization than relative to the CSBs, these observations may have implications for the transcription and maturation of gRNAs. Note that the 5' ends of these gRNAs have not been physically mapped, and that corrections in the predicted editing events may either extend or decrease the 5' and 3' ends of the actual gRNA.

### Guide RNAs predicted using a simple Hidden Markov Model

**Figure 8:**
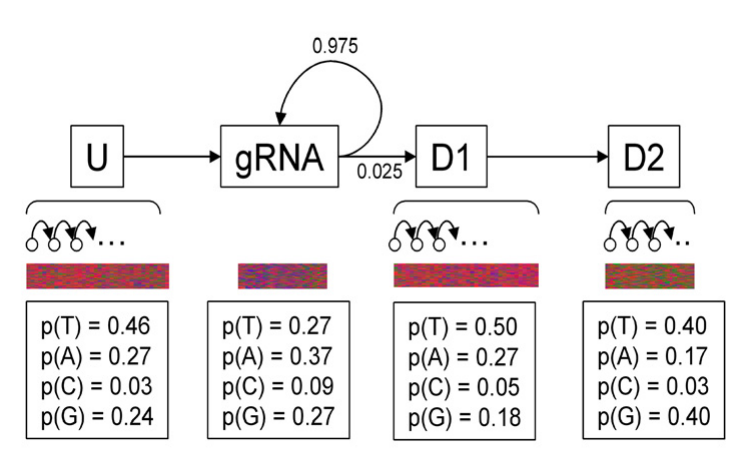
**A Hidden Markov Model for the independent prediction of gRNA genes**. Shown here is the overall structure of the HMM used to predict gRNAs from nucleotide probabilities. The first line depicts the four states: 'upstream', 'gRNA', 'downstream 1' and 'downstream 2', as well as the presence of sub-states for each. The implicit 'START' and 'END' states are not shown. The transition probabilities from the gRNA state are shown, otherwise the transitions are assumed to occur invariably. Depicted below the model is a visual representation of the nucleotide preferences for each state (T-red, A-purple, G-green, C-blue) and the emission probabilities used for each state.

**Figure 9:**
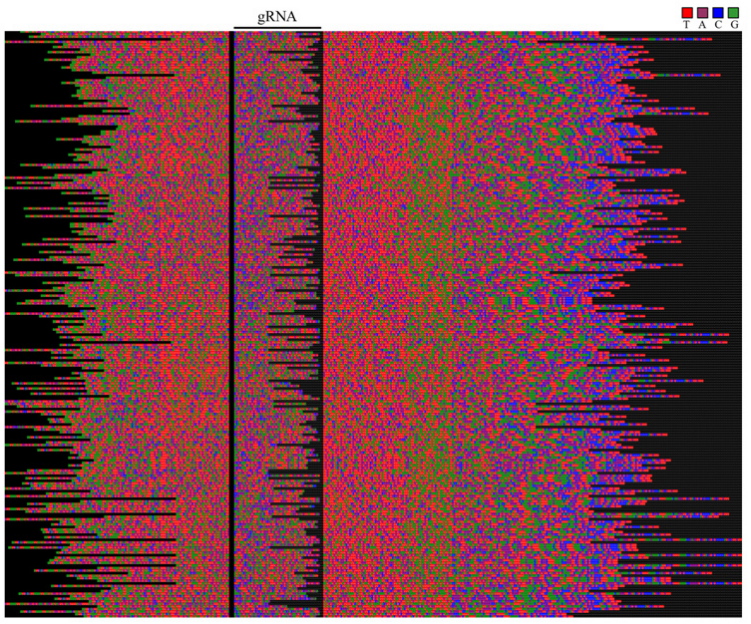
**HMM predicted gRNA alignment**. Shown in the standard spectrogram format used for Fig 7, the gRNAs predicted by HMM for all known *T. cruzi *minicircle sequences are presented, including over 100 sequences for which no gRNA was predicted using local alignments.

Although dozens of gRNAs were predicted using local alignments, approximately half of the minicircle sequences still lacked a statistically significant gRNA-mRNA match. Because the nucleotide preferences observed for assigned gRNAs had a direct correlation to the position of the gene within the minicircle sequence, landmarks for independent gRNA predictions in the absence of known editing events were derived. To do so required a statistical model and a method of finding the optimum path of a sequence through that model. In this case the Viterbi algorithm was used to find the best path through a Hidden Markov Model (HMM) for each known *T. cruzi *minicircle sequence (Fig 8, 9).

The 108 minicircle reads carrying gRNAs predicted by local alignments were first used to test the ability of this method to correctly predict those gRNAs. Although the 5' and 3' ends show differences with those predicted by local alignments, 85% of HMM-predicted gRNAs overlapped the local alignment predictions by at least 20 nt (Additional File 4). The remaining 15% contained very little or no overlap with the local alignment prediction. This method was then applied to all known *T. cruzi *minicircle sequences to predict the gRNAs (Additional File 5) and their locations within each minicircle sequence (Fig 9).

Compared to the local alignment placements (Fig 7), the nucleotide preferences surrounding the HMM predictions were even more visually pronounced (Fig 9). While this was to be expected given the neutrality of the local alignment method to nucleotide usage and the reliance of the HMM on context-dependent usage, there was not enough empirical evidence regarding *T. cruzi *gRNAs to characterize the accuracy of each prediction method. The validity of these predictions will be determined with further experimental editing information. Some of these sequences may also represent pseudogenes.

### Recombination of minicircle sequence classes

Each variable region class was assigned a unique identifier. A single *T. cruzi *minicircle read could contain a string of up to four such class numbers. In general the functional target mRNA of a gRNA had no bearing on its structural location relative to other gRNAs on a minicircle sequence, similar to the situation in *T. brucei*. Recombination among minicircles had not been documented, so this linkage information inherent within the sequence reads was examined for apparently contradictory links between sequence classes.

**Figure 10:**
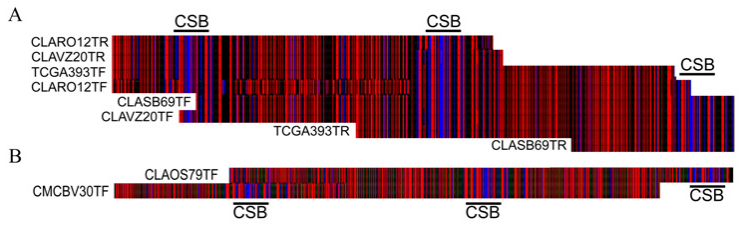
**Recombination of minicircle sequence classes**. A CT 'barcode' spectrogram ('T'-red; 'C'-blue) of CLUSTALX alignment of WGS reads containing identical minicircle sequences. The CSB can be seen here as C-rich regions. Clone CLARO12 displays a combination of variable regions unlike those found on clones TCGA393, CLASB69 or CLAVZ20 and clone CLAOS79 bears a unique combination distinct from that found in clone CMCBV30.

Two examples of discontinuity were found. Reads CLARO12TF and TCGA393TF contain identical downstream variable regions, but the upstream variable regions, separated by the intermediate CSBs, are completely different (Fig 10). The same was true for clones CLAOS79 and CMCBV30. These alternate linkages were evidence of recombination among minicircles.

The minicircle sequences presented represent a fraction of minicircle sequence and as two of 54 CL Brener reads bore evidence of recombination extrapolated to a level of 4% recombinants in the overall minicircle population. However, the minicircle reads presented do not represent complete coverage, and few reads contain three, let alone all four, linked variable regions. This incomplete linkage information is likely to compound the already limited scope of the data, and for these reasons 4% represents a conservative and imprecise estimate for the percentage of minicircle recombinants.

## Discussion

We present here a population study of the minicircles of *T. cruzi *using the identification of 108 minicircle sequences from WGS reads for the CL Brener and Esmeraldo strains of *T. cruzi*, some of which carried evidence of recombination among minicircles. These 108 minicircle sequences, in combination with previously published minicircle and maxicircle sequences, were used to predict 110 total gRNAs using Smith-Waterman local alignments. The positions of these putative gRNAs within the variable region revealed clear nucleotide preferences within and around the gRNAs that were used to create a simple HMM capable of predicting gRNAs from primary minicircle sequence alone. The predicted gRNAs were mapped onto the predicted or experimentally determined sequences of fully edited mitochondrial mRNAs.

The remarkable CSB conservation observed across kinetoplastids [23] is consistent with the observation that there is little intragenomic variability among these sequences. The observation of specific regions of nucleotide preference within the variable region, in particular the ~50-bp relatively 'AT'-rich (and 'C'-poor) region immediately downstream of all predicted gRNAs from minicircle sequences, was unexpected. Given the less linear appearance of this preference when aligned by CSBs, this feature appears to be dependent on gRNA position within the variable region and may be important for some aspect of gRNA transcription or maturation. Although some interesting features of minicircles in other kinetoplastids have been observed, almost nothing is known about the transcription or maturation of gRNAs in any kinetoplastid [28,27]. If the nucleotide preferences were involved in maturation one would also expect to see them surrounding gRNAs discovered on the maxicircle, but this is not the case. This suggests that these sequences are involved rather in minicircle-specific process, although it cannot be excluded that they have no biological importance, merely reflecting the common ancestry of all minicircle sequences. If these sequences are involved in gRNA transcription, it would suggest that maxicircle and minicircle transcription occurs in different manners, using different *cis *signals. The maxicircle gRNAs discovered, specifically the overlap of the *MURF2 *gRNA with the *ND4 *start codon, provide additional evidence that both strands of the maxicircle are transcribed and that the mechanisms of mitochondrial gene expression are likely to be more complex than our current understanding [29].

Because G-U base pairs in complementary RNA strands impart transition tolerance into the editing process, current methods of identifying gRNAs are limited to situations where the fully edited mRNA is known [30] or predicted. The use of permutation tests and false discovery rate control extends the use of local alignments, enabling the robust and systematic identification of gRNAs. The local alignment method used here can tolerate mismatches and gaps that might arise as a result of errant mRNA sequence predictions. It also allows the discovery of pseudo-gRNAs that contain mismatches and have, therefore, lost their functionality. Given the characteristic nucleotide preferences found around the gRNAs, it was possible to develop a method of gRNA prediction that requires no prior knowledge of mRNA sequence.

In addition to many other uses, HMMs have been used to predict genes and alternate splice sites [31,32]. Using the characteristic nucleotide probabilities found at specific distances from gRNAs, an HMM was constructed, representing the first attempt to predict gRNAs independent of known editing events. Although offering useful overlap, these predictions do not always or match exactly the local alignment predictions and how either of these predictions match the actual RNA sequences of the molecules these predictions represent is unclear. In the case of local alignments, only areas of hybridization are detected while other potentially transcribed regions are ignored or areas that are not transcribed are included. While it appears that the HMM is 'missing' or 'adding' sequence compared to the local alignment used to train it, if the transcription or maturation of gRNAs depends on local nucleotide preferences in some way, then the HMM predictions may turn out to be closer than the local alignment predictions. The HMM employed here is extremely simple and with more experimental evidence about gRNAs in *T. cruzi *this model could be further articulated, yielding more accurate predictions. Our HMM is specific to *T. cruzi*, but examinations of *Leishmania *minicircles reveal distinct species-specific regions of nucleotide preference that also align with gRNA position (data not shown). Useful models for these and other kinetoplastids could also be constructed.

Minicircle based assays have been used as markers for *T. cruzi *diversity [33,34]. Based on the gRNA maps, although the resulting protein encoded by the message is highly conserved, the position and sequence of certain gRNAs can be strain-specific. Restriction fragment length polymorphisms could be developed to distinguish these differences, providing another rapid method of determining from which subtype(s) a given DNA sample is from. Further cataloguing and comparison of matching gRNA genes from different strains and DTUs will provide useful information regarding the kinetoplast molecular clock and functional transition tolerance within the gRNAs.

From this minimal dataset two examples of minicircle recombination were identified. Recombination may occur relatively frequently among minicircles, as it does within the DNA of animal mitochondria [35]. The presence of thousands of molecules sharing four conserved sequence regions may enhance even low-level homologous recombination. Computer simulations of minicircle evolution in *Leishmania *show that random segregation can account for the plasticity and frequency of minicircle sequence classes [36], however *Leishmania *minicircles contain only one gRNA each, thereby minimizing the effects of any possible recombination. With evidence of recombination among minicircles, this new dynamic must be incorporated into future models of minicircle evolution in *T. cruzi *[37]. Active recombination may serve as a mechanism to weed out pseudogenes.

Roughly half of all extracted minicircle reads contained a predicted gRNA, and several likely explanations exist for this observation, the first of which is that the predicted editing of *T. cruzi *genes is likely to be incorrect in places, preventing detection of gRNAs that edit those regions. Certainly, the maps for known sequences like ATPase 6 and COIII are more complete than those of other genes. Guide RNAs with small hybridization footprints may also be lost in the statistical noise. The most biologically relevant possibility is that there are probably many variable regions that contain pseudo-guides that once performed a role in editing but have subsequently lost that function. The future sequencing of the edited RNAs for all *T. cruzi *maxicircle genes will aid gRNA identification and validation, add to the incomplete maps presented here, and give insight into the prevalence of defunct variable regions in *T. cruzi *minicircles.

## Conclusion

Incidentally sequenced minicircles were identified among the WGS reads, serving as a reminder that high-throughput data can often have uses beyond that for which the collection methods are designed, and that public availability of this data allows these uses to be discovered. We used these 'contaminants' to glean information about the minicircle population in *T. cruzi *and found evidence of recombination among minicircle sequence classes that suggest a degree of plasticity not explicitly accounted for in many models of minicircle evolution. Together with previously published minicircle sequences, gRNAs were systematically identified, generating maps of RNA editing for many *T. cruzi *maxicircle genes. Although by their nature incomplete, these maps represent a starting point from which to completely characterize the extent of RNA editing in *T. cruzi*. The large number of identified genes allowed the design of a minicircle gRNA prediction model that can locate gRNA genes in the absence of specific mRNA editing information.

## Methods

### Identifying minicircle sequences among WGS reads

In order to identify the intragenomic variability of CSB sequences within the conserved regions, a two-tag discovery strategy was employed using the WGS data from CL Brener and Esmeraldo. The first step was to search restrictively for two tags (CSB-1 and CSB-3 for example) while capturing all of the variability of the third tag between the two landmarks (CSB-2 in the previous example). Employed combinatorially, the intragenomic variability of CSB-1, CSB-2, and CSB-3 was determined. From this information, search parameters were designed to allow positive identification of any number of full CSB-CSB units within any query sequence, without excluding sequences on the basis of slight intragenomic CSB variation. This method was 'greedy' in that if a given sequence contained multiple units it captured all of them at once, thereby preserving any physical linkages between variable regions. To assemble mate pairs with overlapping sequence, a standard overlap alignment algorithm was implemented in the form of a perl script to assemble sequences. Once assembled, the contiguous sequences were each checked by eye using BioEdit. CSB conservation was depicted using Weblogo [38], and spectrograms were constructed using CLUSTALX (alignments), BioEdit (alignment editing and visualization), and Adobe Photoshop (building composites of BioEdit screenshots for spectrograms).

### COIII RT-PCR

A primer complementary to the predicted 3' end of COIII (2 pmol) was added to dNTPs (1 μL of a 10 mM mix) and RNA (1 μg) from CL Brener strain cells in a 12 μL reaction and heated to 65°C for 5 minutes, then placed on ice. For a 20 μL reaction, 4 μL of 5× First Strand Buffer (Invitrogen) and 2 μL of 0.1 M DTT were added. The reaction was warmed to 42°C in a thermocycler for 2 min. and 1 μL of SuperScript™ II (Invitrogen) was added and the tube gently mixed. The reaction was incubated at 42°C for 50 min. PCR was then performed on the RT reaction and a no-RT control using a 55°C annealing temperature. A ~900-bp band seen with ethidium staining represented the full length edited COIII cDNA that was then cloned using a TOPO TA cloning kit (Invitrogen) and sequenced. No signal appeared in the control lane after 30 cycles. Primers used: COIII Forward, TATATTTGTTGGTGTTAGTGG; COIII Reverse, TTATACACACAAATACATAACG.

### Local alignments

The alignment algorithm used was a perl implementation of the Smith-Waterman dynamic programming method of determining the optimal hybridization of a query sequence with a target sequence [39]. Local alignments allow overhangs on the part of the target and query without penalty, ensuring that *in silico *hybridizations of the gRNA with the mRNA could be detected even though the gRNA makes up only a portion of the minicircle query sequence and would hybridize with only a small region of the target mRNA sequence. The only peculiarity of the algorithm used here was the scoring matrix: it was designed to detect complementary (not identity), it allowed for the fact that non-canonical G-U base pairs are common, and it used a large gap penalty, reflecting the expectation that the gRNA should be exactly complementary to the target sequence it edits. For each minicircle sequence, optimal local alignments were performed against the fully edited sequence (predicted or actual) of every edited maxicircle gene and the information from the best alignment was saved to determine its significance (Fig 3A). Although many matrices were tested, the weight matrix used here evaluated a match (A-U, G-U, C-G) and with a score of 4, a mismatch (A-A, C-C, G-G, U-U, A-C, A-G, U-C) with a score of -20, and assessed a gap penalty of 100.

### Probabilities of local alignment scores – permutation tests

In order to determine the probability of a given 'best' local alignment score, the distribution of such scores is required for sequences of a given length and composition against a standard mRNA library. Given these factors this distribution is neither standard nor easy to derive. The method used here to address this problem is the permutation test [40-42] implemented in perl. If a minicircle sequence is permuted, losing all information while retaining the overall nucleotide frequencies, the resulting 'best' score against the mRNA library will be drawn at a certain probability from the unknown but inherent distribution of scores. As the number of permutations sampled approaches infinity, the approximation of that unknown distribution approaches the actual distribution. In this manner the probability of observing a given best local alignment score was empirically estimated using 1000 gRNA permutations (Fig. 3B). Although extremely computationally intensive, this remains the best method of approximating probability distributions that are difficult to derive mathematically.

### Significance of local alignment scores – false discovery rate control

When multiple tests are performed with each allowing a 5% chance of a false positive, the overall number of false positives received can be quite high. Many methods have been suggested to control for multiple testing and several have been broadly applied in biological research [43,44]. The false discovery rate (FDR) method of correcting for multiple sampling [45] was most appropriate for this study given the expectation of discovering exactly one appropriate gRNA match for each minicircle sequence. To perform FDR control (Fig. 3C), where 'X' is a data set including 'n' points, X is ranked in decreasing order of probability (from 0 to 1) by probability such that P(X_n_) > P(X_n-1_)>... X_1_. Then for i = n → 1, where α = 0.05, the following stepwise algorithm is applied: *if at any step i⋅αn>prob(Xi)
 MathType@MTEF@5@5@+=feaafiart1ev1aaatCvAUfKttLearuWrP9MDH5MBPbIqV92AaeXatLxBI9gBaebbnrfifHhDYfgasaacH8akY=wiFfYdH8Gipec8Eeeu0xXdbba9frFj0=OqFfea0dXdd9vqai=hGuQ8kuc9pgc9s8qqaq=dirpe0xb9q8qiLsFr0=vr0=vr0dc8meaabaqaciaacaGaaeqabaqabeGadaaakeaadaWcaaqaaiabdMgaPjabgwSixJGaciab=f7aHbqaaiabd6gaUbaacqGH+aGpcqWGWbaCcqWGYbGCcqWGVbWBcqWGIbGycqGGOaakcqWGybawdaWgaaWcbaGaemyAaKgabeaakiabcMcaPaaa@3E7A@ then k = i. Reject the null hypotheses for all points from X*_1 _to *X*_*k*_. This analysis was performed in perl, with the results visualized by Microsoft Excel (Fig 3D).

### Predicting gRNAs using a simple Hidden Markov Model

HMMs use the known 'emission' probabilities of an observed variable to predict a 'hidden' variable with defined 'transition' probabilities to and from all possible states of that hidden variable. In this case, the differing nucleotide preferences along the minicircle sequence are used to determine the probability that a given nucleotide is positioned within the gRNA state, the hidden state we wish to know. The HMM constructed here consists of four distinct states each with characteristic nucleotide preferences (Fig 8). The upstream (U) state is itself a collection of 40 individual states each with identical nucleotide probabilities and a definite transition onwards to the next sub-state with a probability of 1. After beginning in the 'start' state, a gRNA sequence will enter the upstream state and invariably transition to the gRNA state, the final sequence of which constitutes a gRNA prediction. A nucleotide in the gRNA state can transition back into a gRNA state with a high probability or can transition on to the downstream region which itself is made up of two states, D1 and D2. Like the upstream region the two downstream regions are themselves composed of multiple sub-states with identical nucleotide probabilities, with D1 containing 48 nucleotides and D2 containing 25. The sequences finally leave the model through an 'end' state. The nucleotide probabilities for each state were calculated using the predicted gRNA alignments generated by local alignments (Fig 7). The optimal chain of these states through each minicircle sequence can be recognized as the chain with the highest cumulative probability under the assumption that the probability of transition to a subsequent state depends only upon the nature of the current state and assuming that there is independence among each observed point in the chain. While neither of these assumptions is precisely true for nucleotide sequences, HMMs have been used successfully to predict genes and alternative splicing [31,32]. A perl implementation of the commonly used Viterbi algorithm was applied here to determine this optimal path through each minicircle sequence [46].

## Authors' contributions

ST extracted minicircle sequences, predicted edited sequences of maxicircle genes, performed all analyses of minicircle sequences and drafted the manuscript. IT performed RT-PCR and sequencing of COIII. SW predicted edited sequences of maxicircle genes. NS conceived of the study, participated in data interpretation and preparation of the manuscript. All authors have read and approve of this manuscript.

## Supplementary Material

Additional File 1 Predicted fully edited mRNAs for CL Brener strain of *T. cruzi *(FASTA format). Sequences were generated by manually inserting or deleting Us in the unedited message sequence following the known sequences of the corresponding *T. brucei *edited mRNAs, while preserving conservation of the resulting amino acid sequence. Click here for file

Additional File 2 Text file of predicted gRNAs. File contains predicted mRNA/gRNA hybridizations and scores for those predicted interactions. gRNAs were predicted from minicircle sequences using Smith-Waterman local alignments with predicted mRNA sequences listed in Additional File 1. Click here for file

Additional File 3 PDF file containing maps of edited mRNA sequences showing sites of predicted gRNA associations, also indicated are the various DTUs of the strain from which the sequence is derived. The format of presentation is similar to that of Figure 4, 5, 6, with the gRNA sequences shown below the predicted mRNA sequences. Click here for file

Additional File 4 Image (png format) of HMM test to predict gRNAs obtained by local alignment (LA). Presented in pairs of lines, this spectrogram alignment represent gRNA predictions with the first line representing the HMM prediction and the second line in each pair representing the LA prediction. Line 1 and line 2 therefore represent the HMM and LA gRNA predictions (respectively) for the same sequence, and lines 3 and 4 the HMM and LA predictions for a separate minicircle sequence. A pair of lines is presented for each gRNA sequence with overlapping predictions. Click here for file

Additional File 5 This file lists the most likely gRNA sequence from each *T. cruzi *minicircle sequence as predicted using the HMM. FASTA format. An 85% accuracy is expected for those variable regions that do contain gRNAs. The fraction of variable regions that do not contain a functioning gRNA is not known, and for this reason some of these sequences may represent false predictions. Click here for file
