# Supplementary material for: A population study of the minicircles in Trypanosoma cruzi: predicting guide RNAs in the absence of empirical RNA editing
Source: BMC Genomics. 2007 May 24;8:133. doi: 10.1186/1471-2164-8-133 (PMC1892023; doi:10.1186/1471-2164-8-133)
Supplement: Additional File 3 — PDF file containing maps of edited mRNA sequences showing sites of predicted gRNA associations, also indicated are the various DTUs of the strain from which the sequence is derived. The format of presentation is similar to that of Figure 4, 5, 6, with the gRNA sequences shown below the predicted mRNA sequences. [file 1471-2164-8-133-S3.pdf]

T. cruzi ATPase 6 gRNA map

```
AUGUUUUAUUUUUUUUUUGUGAUUUAUUUUGGUUACGUUUAUUAUUAUGGUUAUAUUAUUGUGUGUGAUCCAGGUUAUGUUUUGUUGUGUAUUUUAAUUGUUUAUUAUUGAUAUUUGAUUUUUUAUUAUUUUGUUUAUUUGA
                    |:|:|:||:|||::|||::|:||||  |:|:|||:|||||:::|
                    AUUGAGUAGAACUGAUGUGAGUAAUCUUGCUAAUGUAAUAGUGC  (AJ748071 Tm)
                                                       ||||:|||:|:::|| ||::|:|::|:|:::||||||:|:||||
                                                       UAAUGACAUAUGUUAAGUUUAGUGUAGAGUGACACAUGAGAUUA  (AJ748041 IIe)
                                                                                                           ||::|:|:|::|:::||:|:||||:||||||||::|
                                                                                                (S80608 I) UAGUUGUGAGUUGGGAAGUGAUAAGACAAAUAAGUU


UUUAUAUUUGUUUAUUGGUUUGUGUUUAUUUUUAUUGUUGUGGUUUAUGUUAUUUAAUUUGUAUAGUUUGAUUUUAUAUUAUUGUAUUACUUAUUUGAAUUUGUAUUUAUUAUUUUGUAUUGUUUUUUGUAUUAUAUGUCGU
|::|||:                                                                                                                          ::|:||||::||::
AGGUAUG  (S80608 I)                                                                                               (AJ748075 Tm)  UGUGAUAUGUAGUG
                         :|| |||:|:||||::::|:||:|::|:|| |:||:|||||||::|:|:||||: ||::||:                                         |:||:||:||:| :|
        (TCKIE52TF IIe)  GAUUAAAGUGACAAUGUUAGAUGCGGUGAACUGAAUAUAUCAAGUUGAGAUAUGUUAGUAUG                        (EMAOB41TR IIb)  AUAUGAUGUAUAAUA
                                 |:|:|::|::|:|||:|:||::||||::||:|||:|:||:|:|:|
                                 UGAUAGUAUUAGAUAUAGUAGGUUAAGUAUGUCAGAUUAGAGUGU  (S80602 I)
                                                      |||:|:::|||:||::|||:||||:||:|::|::|||||:|::||:|||||:||||
                                                      AUUGAGUGUAUUAAGUUAAGAUAUGAUGAUGUGGUGAAUGAGUUUGAACAUGAAUA  (AJ747938 I)
                                                               ||::|::|:||||||::|:|::|||||||||:|:||:|:||||:|||:
                                                               AUUGAGUUGAAAUAUGGUGAUGUAAUGAAUAGAUUUGAGCAUAGAUAG  (TCHRM63TF IIe)
                                                                                               |:||:|:::|| ||:||||::::|||:|:|::|||||:||:|| ||:|
                                                                                (AJ747989 IIb) AUUUGAGUGUACAUGAUAAGGUGUAAUAGAGGAACAUGAUGUAGAGUA
                                                                                                 ||:||:|||::||:|:|||:|||::::|:||||||:||||::||:
                                                                                 (TCGQN08TF IIe) UUGAAUAUAGGUAGUGAAAUAUAGUGGAGAAACAUGAUAUGUAGU
                                                                                               ::||:||::|||:||||||:|:||::::::|:|::||::|||:|||::
                                                                                   (S80639 I)  GUUUGAAUGUAAGUAAUAAGAUAUGGUGGGAGAGUAUGGUAUGCAGUG
                                                                                                     |::||::|:||||:|:| :::|:||:|||||::|:|::||
                                                                                     (AJ748054 IIe)  AUGUAGGUGAUAAGAUACGGUAGAAGAACAUGGUGUGUAG

UUUUAUUUUUAUUUUGUUUUUUGUUGGAUUUUUUUUUAUUUAAUAGUUUAUUGGUUGGUGAUAGUUUUAUGGAUGUUUUUUUUAUCCGUUUUUUGUUAUGUUUUUUGGAAUGUUUUUCUUUAUUGUGUCGUUGUUUGUCAACA
|:
AG (AJ747989 IIb)
:|:||:|::||||:|:|:|:||||||:
GAGAUGAGGAUAAGAUAGAGAACAACU  (EMAOB41TR IIb)
|:|:||::|:||||||:::|:|||||:||
AGAGUAGGAGUAAAACGGGAGACAACUUA  (AJ748075 Tm)
                                          |||:|:||:|:|::::|:|:|::||||:|:||| :|:|:||||||::|:|
                                          UAUUAGAUGAUCGGUUAUUGUUGAAAUGCUUACUGAGAGAAUAGGUGAGA  (TCSPG23TF IIe)
                                                   :::|:::::|||:|:|:|:::|::|::||:|:|||||||:|:|:|:
                                                   GUUAGUUGUUAUUAGAGUGUUUGUAGGAAGAGUAGGCAAGAGAUAG  (S80603 I)
                                                                                       :|||:||||:|::::::|::|||::|||:|||||||::|
                                                                       (TCTCX06TF IIe) UAAAGAACAGUGUGGGGAGUCUUGUAAAGAGAAAUAGUA
```

```
UUUUUACGUUUGUUUUGUAAUUUAUUGUCAUCCCAUUUUUUAUUGUUGAUGUUUUUGAUUUUUUAUAUUUUUUUUUGUUUUUUUUAUUUUUUUUUGUUUUUUAGAUUUUUUAGGAUUUAUUUUAUUUGUUUUUGUGUUUUG
          :::|:|:|||||:||:::|| |  |:||:|:||:|||||::|:|                        ::|||:||:||:|::|::::: :|:||||||
          GUGAGAUAUUAAGUAGUGGUUGUUUGAAGAGUAGCAACUGUAGA  (S80601 I)            UGAAAGAAGUAGAGGAGGGUGUGAGAUCUAA  (AJ748050 IIe)
                                            |:|:|::|::|:|:|:|:::||:|||||:|||||||:|:||::||:                                                 |||:
                                            CGAUUGUAGGAGAUUGAGGGAUGUAAAAGAAAAACAGAGAAGGUAG  (AJ748018 IId, AJ747915 I)     (AJ748017 IId)  AAAU
                                            :||:|::|:|:||:|||:|:|| |:|:||||:||||||:||:||:                                                 :||:|
                                            UAAUUGUAGAGAAUUAAGAGAUUUGAGAAAAGAACAAAGAAGAUG  (EMAKF44TF IIb)               (EMAIL06TF IIb)  UAAGA
                                            ::|||::| ||:|:|:|::|:|:||||:|||::|||: :|:|||:|                                               :||:|:
                                            UGACUGUACAAGAUUGAGGAGUGUAAAGAAAGGACAGCGAGAAUGA  (S80610 I)         (AJ748063, AJ748055 IIe)  UAAGAU
                                                                                                       ||:||||:|||: |:|:||||:||||:|:|||||:::|:|
                                                                                            (S80575 I) AUUUAAAGAAUUAUGAGUAAAGUAAAUAGAAACAUGGAGC
                                                                                                       :|:||:|:|:|: |||:|:||:|::|:|:|||:|||||||
                                                                                           (S80581 I)  GUUUAGAGAGUUAUAAGUGAAGUGGAUAGAAAUACAAAAC
                                                                                                                :|||::|||:|:| ::||| |:|||||:|::
                                                                                                 (AJ748080 Tm)  GAUAGGAUAGAUAUGGACAGAGAACAAGAGU

UUUUUGUUUAUUAUUUUUUGGUUUUUUAUAUUUAUUGGAUUUGUUUGCAGCCAUAUUGCAGUUAUUUAUUUUUUGUAAUAUGAUUUUACAAUUGAUAAUGGAUUUUUUGUUAUUUUUGUUAUUUUGUUAG
|:|:|:                                                     |::|||:||:|:|||||||:||:|||||| |||||||||||:|:
AGAGAU  (S80575 I)                         (AJ748002 IId)  UUGAUAGAUGAGAAACAUUGUAUUAAAAUUUUAACUAUUACUUG
|||                                                                                        |::|:||||:|:|: :::|:||:||::||||::|||:||
AAA  (S80581 I)                                                            (AJ747971 IIb)  AGUUGUUACUUGAGUGGUAGUAGAAGUAAUAGGACAGUC
|:|||:                                                                                      :||:||:::|:|:||::|:||:|||:|||||:|||||:
AGAUAG (AJ748080 Tm)                                                       (EMAH436TR IIb)  GCUGUUGUUUGAGAAGUAGUAGAAAUAAUAAGACAAUU
|::|::|:||::||:|| ::|  :||||||||||:||:||                                                     :||||:::||:|::|::|||:|||:|||| :||||||
AGGAGUAGAUGGUAGAAUGUCUUGAAAUAUAAAUGACUUA  (AJ748063, AJ748055 IIe)           (EMAIL06TF IIb) UUAUUGUUUAGAGGAUGAUAGAAAUAAUAUGACAAUC
:||:|::|:|||:|::|| ::|  :|||||||:||:||:||
UAAGAGUAGAUAGUGGAAUGUCUUGAAAUAUAGAUGACUUA  (EMAIL06TF IIb)
|:||::|::||:||::| :::|:||:||:|::||:|:||||::|
AGAAGUAGGUAGUAGGAUGUUAGAAGAUGUGGAUGAUCUAAGUA  (AJ748017 IId)
```

T. cruzi CR3 gRNA map

```
AAAAAUAAAGAUAUGUAUAAAAUAUAGUAACAAUAuGuuuGAUUGuuuAAuuuuGUUAUUUUuuGuuAuuuGuuuGuACAuuuuuuuuGUUuuuAuuuGuuuGuGAuuuuuGuuuuuAuGuuuAuuAuuuGGuuuuuAuuu
                                   :|:|:|:|:|:|||||:|::||:::|:|:       :||::||||: |::|:|:|:::||||:|:|||:|:||:|
                   (AJ747967 IIb)  UAGAUUGAUAGAUUAAGAUGAUGGGAGAG       UAAGUAUGUGUAGGAGAUAGGGAAUAGAUAAAUAUUAGA  (AJ748044 IIe)
                                                                             |||:|||:||:::||:|:||:::|:|:::|:||
                                                                             UGUGAAAGAAGUGAAGAGUAGGUAGAUGUUGAA  (EMACW52TR IIb)

uuuGuuGGAuuuuuGuuuuuuGuuuAAuAGGGuuuAUUGuuGuGuuuAuuuuuuuuuuuuAuuuuAuCAuuuGAUAuGuAuAuCGAAuuGuuAuuuAuuAuuuAGUUCGUUuAUUAuuAuuAUUuuuAuAA
                                            :|:||:|:||:||||:|:|:|||||||
                                            GAGUAGAGAAGAAAAGUGAGAUAGUAA  (AJ748076 Tm)
```

T. cruzi CR4 gRNA map

```
AUGuAUAGuuuGuGGuAuAuuuuuuuGuuGuuuuGuuuuuuGuuuuuAuuAUUUGUuuGuAuAGGuAuGuuuuAuuuAuuuuGuuAuAGuuGuuuAuuuuuuuuuGuuGuuuuAGGUUGuGAuuuuuuGuuAGUAuuuuGAuu

GuAuAGuuuAuuuuuuuuGuGGCGuuAuAAuuuuGuuuAUuuuuuuuuuuuAuuuuGuuuuAuGUUUUUuAuAuuGUUGUUUUuuuuuGGuuuGuuuGGUuuuuuuuuAuAuuuuuuAUUGuGuuuuAuGuuGuuuuuuAAuu
                                                            ||::|||:|:|: |:::::::||||:|:::|:|||:||:
                                                            AUGUAAAGAGUGGAGUGGUGGAAAAGAGUUAGACAGACU  (AJ748051 IIe)

UAuuuuuuGuGuuGuuuuuuGUuuuGGUUGGuuuuuuuGuuuuuGuuuuuuuuuuuuGUuuAuGuuuAUUuuuGuuuGuAGuuGuuAuuAuuuuAuuGUUUGGGuAGuuAuuuuuAUUUGuGUAuAGGUUUAuuuAuAuGCGu

uuuuuAuuuuAA
```

T. cruzi MURF2 gRNA map

```
AuGuuuGGuuGuuuuAAuuuAGuuuuAuuuuuAuGUUUUGAUUGuAGUCGUGUUUUUGAUUUAUUAUGUGUUAGAGCAUACGAUUUUAUUUUGUGAUGAUUUGAUUUAGAUUAUAUAUUGUAUGAUUUUGUAUUUGAUUUUGU
 ::|||:::|:||:|||||||:|||||||||||||:||:|||
 GUAAAUUGAUAAGAUUAAAUUAAAAUAAAAAUACGAAGCUA (Maxicircle gRNA)
```

GGUAUGCAUAACAUUUAUAUUUAUAUUUAUUUUAGGUUUUUUCGUUAGAAUCUUCUUUAGUUUUGUAUUUGUAUUAUUAUUUAUAACAUUUUUCGGUACAUACACAUUGUCUAUGUUAUAUAUAUACAGGUUACUAUAUAUUU
UACAUAUAUAUAUUAUAUAAUUUCAUAUGUUUCUUUUUUACGUUCGGUAUUAAUUUUCUGAUAUAUUAUUUGGAAUUUUUUACGUUUAUAUUAUUUUAUCUGUUUUUUGAUUUUAUAAGUUUUUCAUGUUUUAUUUAUAAUUU
UUUUGGUCUAUUCUUCACAUUUAAUAUCAUAUUUUGUACGUAUUUGUUUUGCUUAUUUUAUUUUAUAAUUUUUUUUUUAUUCUGUUUUAUAUUUUUUGUAAUACGUUGUUUAUUUGUAAUUGUAUUCGACUUUUUAUUUUUUA
AUUUCGAUAUUUUUAUAUCAUUAACGUUAUGUGAUGUACAGUAUAUAGAUUUCGUGUGUUUCUUAAUAUUGUAUUUUAAUUAUAUAACAAAUUUCAUAUAUGGAUUUUAUUGUUUCAUAAUUAUAUUAGGGUUAAUUUUUUUA
UUAUUAUACAUGGUACUUAAUUUAUAUUUUGGUUUUUUUUUUAUAAUUUAUGGAUUAAUAUCUUUUCUGAUAAAUUAUAUAUUCUGAAUAUAUAUAAUAUAUAGUAGAAGUUGUUAUAUAUUAAUGCCGUCUAUUUUAAUAUU
UUUUAAGUUUAUUUAUUUUGAUGUUUUUUUAAUAUUUAUAUUUAUAUUAAUUUUGUUCAUAAUAUGUUUUUUUAGUUUUUUUUUAAAAGAUUUUUUAUUUUUGUCAUUGUUUUUUGAUAUAUUUGGAGCACUGUUUAAUUACG
AUAUAUACACAUACUCUGUAUUUUAUUACCAUUUACACCAAUUUUGUGUAACGCAAUUAUUAUUUCUUUAUACGUAG

T. cruzi ND3 gRNA map

```
AuGAAUuCACGuuuAGUGuuuuAuACAuuAGAuuuGuGuuuuGuuAGuuGuuuAuuuuuuGuGuuAuuGACUACCAuuAuuuGuGuuuuGUUAuuuAUuuuuAuGAUAAUGuuAuuuuGuuuuuAuuGuAAuuuuuuGuuuuu
                                    ::::|:|:::|::|||:|:|||:||||||||
                 (TCKFC46TR IIe)    UGUGAGAUGGUGGAUAGAGAUAUUAUUACAA

AUGGUUUuuGuuuuAuAuuGuGuGuAuAGGGUGUUUAuGGuAuuuuuGGGAuCCAGuAUAUUUGUGuGAuAuCUUAuuAUuuuGuuuAuGGuGUUUUuuGuuAuAuuGUAUUUUGUUUGAAUUCUUAAAAUAA
```

T. cruzi ND7 gRNA map

```
AuGuuAuuuuuGGuGGuuuuuuuACAuuuAuAuCGUuuuACAuuuGGUCCGCAGCAuCCCGCAGCCCAUGGuGuuuuGuGuuGuuuAuuAuAUUUUuGUGGuGAAuuuAuuGuuuACAuUGAUUGuAuuAuAGGUUAUUUGCA
UCGUGGUACAGAAAAGUUAUGUGAAUAUAAAACAAUAGAACAGUGUUUGCCGAUUUuUGAUAGGUUAGAuuAuGuuAGuGuuuGuuGuAAuGAACAuuuGuuGuCuuuAuGuuuuGAAuAuAuGuuGCGGuGuuAuuuGuCAu
                                                      |||::|:|:::||:|::||:|||:||::|||||:||:
                                                      AAAGUUGUUUGAUUUGGUAUAAUUACGGACAACGUUG  (Maxicircle gRNA)

UACGuuGuGCAuuuAuGCGuuuGuuAAuuGuAGAAuuuACCCGuGGuuuuAAuGGuuuAuuAuGuAuAuCAuGUAuGGuuuuGGAuuuuGGuuGuuuGACUCCAuuAuuAuGAuCAuuuGAGGAACGUGAUAAGuuGAuGACA
                                                            ||::||||:||:::||::|:||::|:|:
                                                            AUGUAUAUGGUGUGUAUUAGAAUUUGAG  (EMAGB04TR IIb)

uuuuuuGAuuuuuGUUGuGGuuGGCGuAuGCAuuuAGCUUUCAuGGuuuuAuuGGGuACUCuuGAuGAuuuuGuuuuuGGuuuuGuuGAuuuuuuAuuAuuAuUGAuAAuAuCGuGuuuGuuuGuuAuGGAuuGuuAUGAuuu
                                            ::||:||:|::::||:||:||::|:|||:||||::||||::|::||
                                            UUAAGAUGAUUUGUGGGAGCUGUUGAAAUAAAAGUCAAAGUAGUUA  (EMABQ94TF IIb)
                                                                                                              |||:|:||::|:|:::|:::|:|| |||:|:|:
                                                                                              (AJ747982 IIb)  UAGUAUAAGUAGAUGGUGUUUGACUAUAUUGAG
                                                                                                             ||||:|:|:|:|:::|:||::|:|:|:|::|:||
                                                                                             (AF242563 FGz)  AUAGUAUAGAUAGGUAGUAUUUGAUAGUGUUGAA

GuuGuuuGuGGGuAAUCGuuuGuuuuAuuuGCGuuuACGuGGuuuAuCGuuuuuuGAUUuGuAuGAuUUGGuuuUUAAUAGuuuAAGuGGuGuuuuGuCuCCuGCAuuAGGuAuGGuAuGGGAUuGUCGuuuAuuuAGuuGuu
:||||:|||::|||
UAACAGACAUUCAU (AJ747982 IIb)
|:|||:| ||:||||:
CGACAGAGACUCAUUG (AF242563 FGz)

UUGAAUuAUAUUUUAuGuuuuGuuAuGAuuAuuGuuuuuGuuuuAuAGGuuAuGCGuuuGAUCGuuuAuuuuuACGuuuAuuuGAuAUGCGuAuGAGuuuGuuAAUUUGUAAACAAuGuuuuuuuGuuGGuuuuuuuGUUUUU
                                                                                                                                    |::|:::|||:
                                                                                                                   (EMABQ94TR IIb)  AGGAGUGAAAG

GGuuuuGuuuGuuuGuuuGAuuAuuuGuAuuGuGAUAuAACCAuuGAGACCAuuAuuAuGuuGuuuuAuAGuuuGuGAuGuuGuuGuuuACCCGGuAuAUCAuuuGCUUGUGuuGAACACCCCAAGGGuGAGuAUUGuuuGuu
:::|:|:||:: :|||:|:||:                                                                                                               ||::|:|::|
UUGAGAUAAGUUGACAGAUUAG (EMABQ94TR IIb)                                                                              (EMAJK77TR IIb)  UAGUAGAUGA
          :|:|:|:|:|:|||:|:||:|:|||||:|||||:|:||||
          UAGAUAGAUUGAUAGAUAUGAUACUAUGUUGGUGAUUCUG  (AJ747931 I)
                                         ||:||||| |||||:||::::::||||||||:|:|:|::|
                                         GUGACUCUUGUAAUGAUGUGGUGAAAUAUCAGAUAUUGUA  (EMAAQ84TR IIb)

AuuGUGuuuuuGuGUuGGuuuAuGUUCCCGuuuACGuuuACGuuGuGCGGAuuuuuuGCAUAUuuGuuuGuuAGAuGuuuGuuuACGuGGuuuuuuAuuGCAuGAuuuAGuuGCCGuuuuGGGuAAuAuUGAuGuuGuuuuuG
|:|:::||:|||:::|:||:|||||||                                        |::|||: ::|:::|:|||::::: ||:||:|:||::|::
UGAUGUAAGAACGUGAUCAGAUACAAG  (EMAJK77TR IIb)                       AGUAAUUAGUAGGUAGAUGUGUUGUAAGAUGAUGUGUUGG  (AJ748000/8016/8022 IId)
                                                                ::||::|:||||::|:::|:||:|:|||:||||:||||:||:
                                                                GUAAGUAGUCUAUGAGUGAGUGUAUCAAGAAAUGACGUGCUG  (AJ747960 I)
                                                                  |:|:|:|||::|:|:::|||::::|:||||:|||||:|:
                                                                  AGAUAGUCUGUAGAUGGAUGUGUUGAGAAAUGACGUAUUG  (AJ748059 IIe)
                                                                                             |:||:|:|||:|:|:|::|:||:|::
                                                                                             AGAUGAUGUAUUGAGUUGAUGGUAGG  (AJ748034 IId)
                                                                                                                                |||||::|::|::||
                                                                                                               (EMABQ94TR IIb)  AACUAUGAUGAGGAC

GAuCCGuAGAUCGuuAG
:|: |:||||||:|:||
UUGUGUAUCUAGUAGUC (EMABQ94TR IIb)
```

T. cruzi ND8 gRNA map

```
UuGuuuuuuuuuGAuuuuuuAUUUUUuuuuuuuGuuuGuuuuuAuAuGuGUUUuGuuuGuuGuGuuACCAUUUGUUuACCCAuuGAAuuAACAAuuGuuAGuuuAuuAGUuCCuGGuGAACAuuuuuuGCGUUUUuAUUGGuG
                                                                                                                                      |:|||::::
                                                                                                                     (AJ747988 IIb)  AGUAAUUGU


uGGuuuAGAACGuuGuAuuGCuuGuCGuuuAuGuGAuuuGGUUuGCCCuAGuuuAGCAuuGGAuGUUCGuGuuGGAuAuuuAAGuuuuGGuGGCCACCGuuuuGCCGAuuGUUUAACAuuAAGuuACCGCCGuuGuAuuuAuu
|:::||:||||:||:|||::|                                                                                                              ::|:|||:|||:
AUUGAAUCUUGUAAUAUAGUG  (AJ747988 IIb)                                                                             (EMAEC50TF IIb)  UGAUAUAGAUAG


GuGGuuuuuGuAuGCAuGuuuGCCCGACGGAUGCCAuuACACAUUCAuuGuuuGuuAuGuGuuuuuGuuGuuuAGCCAUGuAuuuAuuGGCACCCAAGuuuuuAuuGuuuGGuuGuuGuuuuAuGuuAuuuGAuuuuuAuuuG
:|::::|:|||||:||:::                          |||:::||:|:|::::::||::|::|:||||||||||:|:||
UAUUGGAGACAUAUGUGUG  (EMAEC50TF IIb)         GUAGUGAAUAGUGUGUGGAAGUAGUAGAUCGGUACAUGAGUA  (AJ747930 I)
                                                                                       |::||| |||::|:||||:::|||:|::||||:|||
                                                                                       AUUGUGUGUUUGAGAAUAGUGAACUAGUAACAGAAU  (EMACZ60TR IIb)
                                                                                                            |:|::||:|:::|||||::|||:|||:|:||||:::
                                                                                           (EMAD906TR IIb)  AGAUUAAUAGUGAAAUAUGAUAGACUGAGAAUAGGU
                                                                                                              :::|::||:||:||::::|||:: : |:|||:||
                                                                                                 (AJ747961 I)  GUUAGUAAUAAGAUGUGGUAAGUAGUAGAUAGAC
                                                                                                                        ::||:::|:|||:||||::|:|||
                                                                                                     (EMANP33TF IIb)  GUAAUGGAGUACGAUAAGUUGAAA



uGuuuuGuGuAG
||||:||
ACAAGAC  (EMAD906TR IIb)
|||:|::
ACAGAGU  (EMANP33TF IIb)
||||
ACAA  (AJ747961 I)
```

T. cruzi ND9 gRNA map

```
AuGuuuAuAUuuuuAuGuuuAUUuCGuuuAuGuuuuuGuuuAAUUUUAuuuuAUUGuuuGuGuuGuAGAuGAUGUUUGuuuGuuuuGuuGAuuGuAGUuuuuuAuuuuuuuAuuGuuuuGuuAGuuuuuuuuGuuuuAUUG
                                                                                  :|:||:|: |:|::|::|||:|||::|:|| ||:|:|:||||:|:
                                                                  (EMAGF40TR IIb) UAGAAUAGAUGAUGUUGAAAGAUAGGAGAACAAUAGAGCAAUUAG
                                                                                                            :||:|::::||::::|:|:||||:||:::|:||||
                                                                                           (EMALF94TR IIb)  GAAGUGGUGAAGUGGUUAGAAAAGAAUGGAGUAAC
                                                                                                              :|||:::|||:|:|:::||:|::|:||:|||||
                                                                                                (AJ748081 Tm) GAUAGUGAAAUAGUUGGAAGAGGAUAAGAUAAC

UAUGuuuuuAUUUUuuAAuuuGUGGuuuuuAUUUUuGuAUUGUuGuGAUUUGuuAuuGGuuGAuuuuuGuGUGUUUuGuuuuuGuCGuuuuAuGUUGuuGuAuAuuuuAuuuuGuuuAuuuuuGUGuAuuCGuuuGuGuuuuG
||::|:|:|||
AUGUAGAGAUA  (EMALF94TR IIb)                                                                                 |:|::||:|:::|:|:|::|||:::|:||::|:|||||||:
||:||:|:||:                                                              (TCGRC69TR IIe) AGUGUAAUAGUGUGUGAGGUAAGGUAGAUGGAGACACAUAG
AUGCAGAGAUG  (AJ748081 Tm)

UUUuGuGUuGuuuGuuuuuAuuuuuuGGGuuGuuuuuuAGuuuuAGuuGuuuuuGuuAuACAUUUuuAUUAUUAGAACGuGAAuGuuuuGAUUUGuuuGGUUUuuAuuuuGUUGGuAAuGAuGuuuuACAUCGuuuAuuuGuu
                                                                           |||:|:||::||:|:|:||:|:|::::|:
                                                                           UUGUAUUUGUAAGAUUGAAUAGAUUGGAG  (AJ747984 IIb)

GAuuGAUuuuuuGuuGGuuuuuuuuuGuuGAAAuGuuAuCCGUUAUUUGUGUUAuuuGuGuuAuuAuuuuGuAuAuuAuuGGAGGAAAUAAuAUGuACAuuuACAAuGuuGuuuuuGuuGuuGCAuACCAAuuuuuAuuuACA
UuAuuuuAUGUAA
```

T. cruzi RPS12 gRNA map

```
AUGuuuuuuuuGCGuAuGuGAUUUUUGuAuGGuuGuuGUUuACGUUUuGuuuuGuuuGuuuuAuGuuAuuAuAuGAGCCCACGAuuACCCAGUUCCGGuAACCGUCGuGuGuuAuAuGCCGuAuuuuAUUUGuAuAGuuuuGu
                                                                                                             ::||:||:| :||:|:||||::||:|::|:::|:
                                                                                               (AJ747962 I)  UGAUGUAUGAUAUGAGAUAAGUAUGUUGAGGUAG


UuGGuGuGGCGUUUGuuuuuuuuGuGGuAuuuuuAuAuuGuCAuuuGCAuuAuuuAuuAuAGAAGGuGGuGGuuuuAuuGAuuuACCCGGAUAuAAAAuAUUAuCACGUAuuuGUUAuuAG
:|::|:|||:|||::|
GAUUAUACAUGCAGUA  (AJ747962 I)
    |||:|:|:||::|:|:||:::::|||:||||||:|||||:|
    ACAUAUGUAAUGAGAGAAGUGUUAUAGAAAUAUGACAGUGA  (AJ747970 IIb)
                                                  ||:||||:|:||||::||:::::|||||::|||:|||
                                                  AUGAAUAGUGUCUUUUACUGUUGAAAUAGUUAAGUGG  (EMACW52TR IIb)
```
