# Supplementary figures and images for: A population study of the minicircles in Trypanosoma cruzi: predicting guide RNAs in the absence of empirical RNA editing
Source: BMC Genomics. 2007 May 24;8:133. doi: 10.1186/1471-2164-8-133 (PMC1892023; doi:10.1186/1471-2164-8-133)

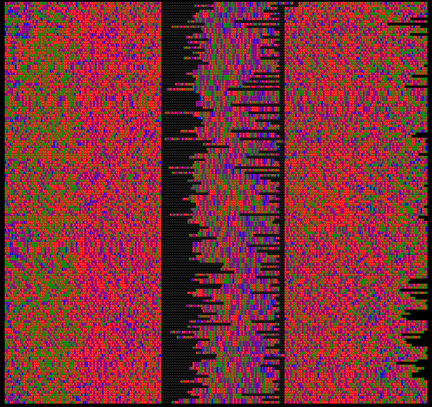

Supplement: Additional File 4 — Image (png format) of HMM test to predict gRNAs obtained by local alignment (LA). Presented in pairs of lines, this spectrogram alignment represent gRNA predictions with the first line representing the HMM prediction and the second line in each pair representing the LA prediction. Line 1 and line 2 therefore represent the HMM and LA gRNA predictions (respectively) for the same sequence, and lines 3 and 4 the HMM and LA predictions for a separate minicircle sequence. A pair of lines is presented for each gRNA sequence with overlapping predictions. [file 1471-2164-8-133-S4.png]
